# Impact of metabolic syndrome on bone mineral density in men over 50 and postmenopausal women according to U.S. survey results

**DOI:** 10.1038/s41598-024-57352-z

**Published:** 2024-03-25

**Authors:** Mo-Yao Tan, Si-Xuan Zhu, Gao-Peng Wang, Zhong-Xing Liu

**Affiliations:** 1 Chengdu University of Traditional Chinese Medicine, Chengdu, Sichuan China; 2 Dujiangyan Traditional Chinese Medicine Hospital, Chengdu, Sichuan China

**Keywords:** Metabolic syndrome, Bone mineral density, National Health and Nutrition Examination Survey, Males over 50 years and postmenopausal females, Diseases, Medical research, Risk factors

## Abstract

Metabolic Syndrome (MetS) and bone mineral density (BMD) have shown a controversial link in some studies. This research aims to study their association in males over 50 and postmenopausal females using National Health and Nutrition Examination Survey (NHANES) data. Postmenopausal females and males over 50 were included in the study. MetS was defined by the National Cholesterol Education Program Adult Treatment Panel III guidelines. BMD values were measured at the thoracic spine, lumbar spine, and pelvis as the primary outcome. Weighted multivariate general linear models have been employed to explore the status of BMD in patients with MetS. Additionally, interaction tests and subgroup analyses were conducted. Utilizing the NHANES database from 2003 to 2006 and 2011–2018, we included 1924 participants, with 1029 males and 895 females. In postmenopausal women, after adjusting for covariates, we found a positive correlation between MetS and pelvic (β: 0.030 [95%CI 0.003, 0.06]) and thoracic (β: 0.030 [95%CI 0.01, 0.06]) BMD, though not for lumbar spine BMD (β: 0.020 [95%CI − 0.01, 0.05]). In males over 50 years old, MetS was positively correlated with BMD in both Model 1 (without adjusting for covariates) and Model 2 (considering age and ethnicity). Specifically, Model 2 revealed a positive correlation between MetS and BMD at the pelvis (β: 0.046 [95%CI 0.02, 0.07]), thoracic spine (β: 0.047 [95%CI 0.02, 0.07]), and lumbar spine (β: 0.040 [95%CI 0.02, 0.06]). Subgroup analysis demonstrated that the relationship between MetS and BMD remained consistent in all strata, underscoring the stability of the findings. In postmenopausal women, after adjusting for all covariates, a significant positive correlation was observed between MetS and BMD in the pelvis and thoracic spine, whereas this correlation was not significant for lumbar spine BMD. Conversely, in males, positive correlations between MetS and BMD at the lumbar spine, thoracic spine, and pelvis were identified in Model 2, which adjusted for age and ethnicity; however, these correlations disappeared after fully adjusting for all covariates. These findings highlight the potential moderating role of gender in the impact of MetS on BMD.

## Introduction

Bone mineral density (BMD) refers to the quantity of minerals in bone tissue and is a measurable indicator of bone mass and strength^1^. When BMD drops below a certain threshold, it can lead to osteoporosis and increase the risk of fractures^2^. It has been estimated that 46% of Americans aged 46 and above have low BMD^3^. Alarmingly, the economic impact of fractures associated with osteoporosis is substantial, with an annual cost of approximately $17.9 billion in the United States^4^. Bone loss progresses silently and gradually, with symptoms typically not emerging until the occurrence of a devastating fracture^5^. Therefore, understanding the factors that impact BMD is of utmost importance. Some elements in daily life can affect BMD, such as intake of fatty foods and exercise^6,7^. It has been suggested that elevated levels of high-density lipoprotein cholesterol (HDL-C) may influence osteoclast activation or function by activating inflammatory responses^8,9^. Notably, exercise is known to enhance the improvement of bone tissue and increase the load on bone tissue, thereby promoting the necessary stresses on cellular processes such as osteoblasts, osteoclasts, and osteocytes, which can result in significant changes in BMD^10^.

Metabolic Syndrome (MetS) is a cluster of cardiometabolic risk factors, including central obesity, elevated triglycerides, high blood pressure, elevated fasting glucose, and low levels of HDL-C^11^. In Western countries, its prevalence among adults is estimated to range from 20 to 25%^12^. Significantly, this rate increases with age, reaching 40% to 45% in those aged 50 and above^12^. Specifically, in Germany, Spain, and Italy, the economic impact on the health system attributed to MetS in patients with hypertension is estimated at €2.4427 billion, €190 million, and €487.7 million, respectively^13^. One study highlights that MetS significantly contributes to the burden of non-communicable diseases, posing an escalating public health challenge for developed and developing nations^14^.

Men over 50 and postmenopausal women are at a heightened susceptibility for MetS, and this particular demographic also exhibits increased sensitivity to changes in BMD^15^. This correlation can be attributed to osteocalcin, a protein that reflects the activity of osteoblasts and is responsive to BMD^16^. Previous research has indicated a decline in serum osteocalcin levels in both men and women after age 50, implying a significant association between age and BMD alterations^17^. Furthermore, there exists a negative correlation between serum osteocalcin levels and the risk of MetS^18^, suggesting that as serum osteocalcin decreases with age, the likelihood of developing MetS also increases. Based on the evidence presented above, it is clear that this group has significant research value.

Since 2005, research has started to investigate if MetS characteristics could heighten the risk of non-vertebral fractures, uncovering that certain aspects of MetS may help mitigate this risk^19^. By 2010, enhanced research methodologies and the utilization of big data led Park et al. to explore the connection between MetS and BMD in postmenopausal women, establishing a positive correlation between them^20^. Concurrently, Szulc et al. delved into the association between MetS and bone health in older men, finding the impact of MetS on BMD to be negligible at that time^21^. From 2020, as cross-national and multicentric studies grew, the scope of research broadened to encompass various ethnicities and regions, shedding light on the genetic and environmental influences on the MetS–BMD relationship^22–24^. Beginning in 2021, the focus has shifted towards understanding how MetS could affect BMD through mechanisms like the influence on inflammatory markers and hormone levels^25–27^. Over the past 2 years, studies have also been underway to examine how dietary interventions for MetS patients could enhance BMD^28,29^.

In summary, the effect of MetS on BMD continues to be a contentious topic, especially as limited research has directly addressed the aging population (men over 50 and postmenopausal women) to assess the link between the two. To address this research gap, we analyzed data from the National Health and Nutrition Examination Survey (NHANES) spanning 2003–2006 and 2011 to 2018. We applied weighted multiple linear regression, subgroup analysis, and interaction tests to further explore the relationship between MetS and BMD in males over 50 years and postmenopausal females.

## Methods

### Data available

The NHANES is a representative, cross-sectional survey collected over several years that provides extensive information on nutrition and health status for U.S. adults. The data is collected uniformly every 2 years as part of a multistage process and is managed by the Centers for Disease Control and Prevention (CDC). Participants have given written informed consent to participate in the NHANES program, which has been approved by the Ethics Review Committee of the National Health Statistics Research Center^30^. Surveys and data from the study are available on the NHANES website (http://www.cdc.gov/nchs/nhanes/). This study strictly adhered to the Strengthening the Reporting of Observational Studies in Epidemiology (STROBE) principles for cross-sectional studies^31^.

### Study population

**Figure 1:**
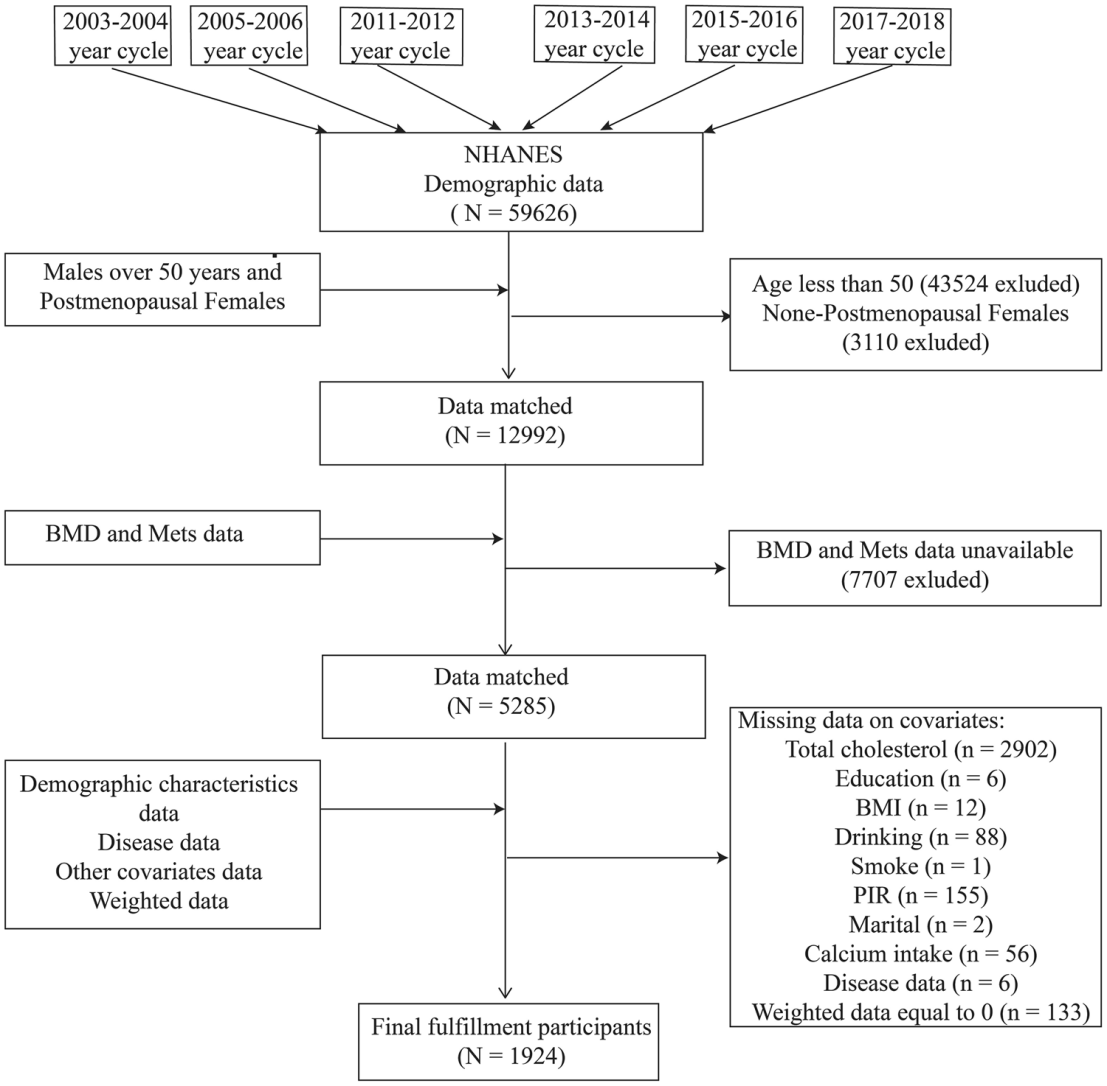
Flowchart of the sample selection from the National Health and Nutrition Examination Survey (NHANES).

Our study analyzed data from six NHANES survey cycles: 2003–2004, 2005–2006, 2011–2012, 2013–2014, 2015–2016, and 2017–2018. The exclusion of the 2007–2008 and 2009–2010 cycles was due to the NHANES's omission of lumbar spine, pelvis, and thoracic spine BMD measurements during these periods. Our initial dataset comprised 59,626 participants across the selected 2-year cycles following a meticulous screening process. Initially, individuals under 50 were excluded, amounting to 43,524. Subsequently, non-menopausal females were eliminated, totaling 3110 individuals. Additionally, participants needing BMD or Mets data, amounting to 7707 subjects, were also excluded. Other exclusions comprised individuals missing total cholesterol data (n = 2902), educational level information (n = 6), Body Mass Index (BMI) data (n = 12), drinking status (n = 88), smoking status (n = 1), poverty income ratio (PIR) (n = 155), marital status (n = 2), calcium intake (n = 56), disease data (n = 6), and those with a weighting of zero (n = 133). After this comprehensive screening, a final sample of 1924 subjects remained eligible for analysis, as illustrated in Fig. 1.

### Menopausal status

Menopausal status was assessed through a self-reported reproductive health survey. Women who reported not having any menstrual periods in the last 12 months in response to the question “Have you had at least one menstrual period in the past 12 months?” and indicated either “hysterectomy” or “menopause/change of life” as the reason for the absence of menstruation were classified as postmenopausal. Further information on the reproductive health questionnaire can be found on the NHANES website^32^.

### Definition of MetS

In this study, MetS was considered as an exposure variable. A MetS group was defined as those who met at least three of the following criteria according to the National Cholesterol Education Program Adult Treatment Panel III guidelines^33^: (1) triglyceride levels above150 mg/dL; (2) Men with a waist circumference of 102 cm or women with a waist circumference of 88 cm; (3) A level of high-density lipoprotein in men and women should be at least 40 mg/dL or 50 mg/dL, respectively ; (4) blood pressure ≥ 130/ ≥ 85 mmHg; and (5) fasting glucose ≥ 110 mg/dL. The secondary outcomes are as follows: Waist circumference was divided into two groups, namely low (< 102 cm in men or < 88 cm in women) and high (≥ 102 cm in men or ≥ 88 cm in women) categories. Triglyceride levels were also categorized as low (< 150 mg/dL) and high (≥ 150 mg/dL) groups. High-density lipoprotein levels were classified as low (< 40 mg/dL in men or < 50 mg/dL in women) and high (≥ 40 mg/dL in men or ≥ 50 mg/dL in women) groups. Blood pressure levels were classified as low (systolic blood pressure < 130 mmHg and diastolic blood pressure < 85 mmHg) and high (systolic blood pressure ≥ 130 mmHg or diastolic blood pressure ≥ 85 mmHg) groups. Lastly, fasting plasma glucose levels were categorized as low (< 110 mg/dL) and high (≥ 110 mg/dL) groups for analysis.

### BMD measurement

This study considered thoracic, lumbar, and Pelvic BMD as outcome variables. APEX 4.1 was used to analyze DXA scans and measure BMD using a dual-energy X-ray absorption scanner (Hologic Discovery DEXA Scanner, Hologic, Inc., Bedford, MA, USA)^34^.

### Covariates

We identified potential confounding variables for the correlation between MetS and BMD using the multivariable adjustment models used in previous studies^35–37^. The demographic variables examined in our study encompass gender (male/female), age (in years), ethnicity (Mexican American/Non-Hispanic white/Non-Hispanic black/Other races), educational level (less Than 9th grade/9-11th grade (includes 12th grade without diploma)/High School Graduate/GED or Equivalent/Some College or AA Degree/College Graduate or above), marital status (married/widowed/divorced/separated/never married/living with partner), PIR (low-income/middle-income/high-income)^38^, total cholesterol (mg/dl), low-density lipoprotein cholesterol (LDL-C) (mg/dl), BMI (kg/m^2^), and calcium intake data is collected through the first 24-h dietary recall conducted by participants. This study employs questionnaires to delineate and precisely categorize the following variables: Smoking: Questions such as "Do you now smoke cigarettes?" and "Have you smoked at least 100 cigarettes in your lifetime?" classify smoking status into never, former, or current smokers, following methodologies detailed in a preceding report^39^. Alcohol Consumption: Assessment of alcohol intake is based on responses to "Have you had at least 12 alcoholic drinks in your lifetime?" and "Have you had at least 12 alcoholic drinks in the past year?" supplemented by ALQ130, which estimates the average number of alcoholic drinks consumed per day in the past 12 months. This classification of alcohol consumption patterns into never, former, heavy, mild, or moderate is consistent with criteria established in an earlier report^40^. Stroke (Yes/No): Confirmation of stroke history is ascertained through participant affirmations to “MCQ160F: Ever told you had a stroke?” or “SPQ070D: Ever told had a stroke?”. For accurate identification of hypertension and diabetes, a combination of laboratory data and questionnaires was employed. For diabetes, various markers, including fasting blood glucose, random blood glucose, 2-h oral glucose tolerance test (OGTT) blood glucose, and glycohemoglobin HbA1c were utilized. Questionnaire components asked about a doctor's diagnosis of diabetes, taking diabetic pills to lower blood sugar and current insulin use. Diabetes was defined based on the following criteria: a doctor's diagnosis, glycohemoglobin HbA1c levels of 6.5% or higher, fasting glucose levels of 7.0 mmol/L or higher, random blood glucose levels of 11.1 mmol/L or higher, 2-h OGTT blood glucose levels of 11.1 mmol/L or higher, or the use of diabetes medication or insulin^41^. For hypertension diagnosis, both blood pressure measurements (systolic/diastolic pressure) and questionnaire surveys asking "Ever told you had high blood pressure" and "Taking prescription for hypertension" were used. Hypertension is defined as taking antihypertensive medication, a doctor's diagnosis of hypertension, or having a systolic blood pressure of ≥ 140 mmHg or a diastolic blood pressure of ≥ 90 mmHg on three consecutive readings^42^.

### Statistical analysis

In our study, we rigorously adhered to the statistical analysis protocols prescribed by the CDC. In the NHANES study, sampling weights, stratification, and clustering methods were applied to accurately reflect the complex multistage sampling design of the representative noninstitutionalized U.S. population and to ensure the precision of statistical significance estimates. According to NHANES official guidelines, when selecting weights, priority is given to variables involving the smallest population group. Following this principle, the study utilized Mobile Examination Center (MEC) examination data that included fasting triglyceride data and, as recommended, selected the corresponding subweight (WTSAF2YR). In line with NHANES analysis guidance, new sampling weights for the combined survey cycles were created by dividing the 2-year weights for each cycle by 6. Continuous variables were presented as mean with standard error, while categorical variables were expressed as percentages. Subsequently, we employed a weighted Student's t-test (for continuous variables) or a weighted chi-square test (for categorical variables) to evaluate differences between groups. Weighted multivariate linear regression was utilized to examine the relationship between Mets and BMD among males over 50 years old and postmenopausal females. The data are presented in terms of coefficients (β) and Confidence Intervals (CI). To guarantee the precision of the findings, confounding factors were considered. In Model 1, no covariates were accounted for. Model 2 incorporated adjustments for age and race. Model 3 included adjustments for age, race, education level, PIR, marital status, BMI, smoking status, alcohol status, calcium intake, LDL-C, total cholesterol, diabetes, stroke, and hypertension. To ensure the stability and reliability of our research findings, this study carried out subgroup analyses separately for male and female groups. During this process, we meticulously considered multiple stratification factors, including age, BMI, smoking status, drinking habits, hypertension, stroke, and diabetes status. The comprehensive consideration of these factors aims to delve into their specific impact on the research outcomes, thereby verifying the universality and stability of our findings across different population subsets. Additionally, interaction tests were performed to assess potential interactions among these variables.

A significance level of less than 0.05 is typically considered statistically significant in statistical analysis. The statistical analyses were conducted using R software (version 4.1.2; http://www.R-project.org, R Foundation for Statistical Computing, Vienna, Austria).

### Ethics approval and consent to participate

Ethical review and approval were exempted for this study as it made use of publicly accessible data sourced from the National Health and Nutrition Examination Survey (NHANES) database. Authorization for the use of the NHANES database was granted by the National Center for Health Statistics (NCHS) in the United States. The study protocols underwent approval by the NCHS Research Ethics Review Committee, and all participants in the NHANES survey provided informed consent.

## Results

### Characteristics of participants stratified by metabolic syndrome status

Table 1 presents the baseline characteristics of the study participants based on their MetS status. Among the total of 1924 participants, 40.62% were identified as having MetS. The MetS group had a mean age of 59.34 ± 0.36 years, with 54.19% being male and 45.81% being female. The lumbar spine BMD was measured at 1.04 ± 0.01 g/cm^2^, the pelvic BMD at 1.26 ± 0.01 g/cm^2^, and the thoracic spine BMD at 0.88 ± 0.01 g/cm^2^. The remaining 1099 participants belonged to the non-MetS population, with a mean age of 58.67 ± 0.32 years and a male-to-female ratio of 52.00 to 48.00. The lumbar BMD measured 1.00 ± 0.01 g/cm^2^, while the pelvic BMD measured 1.20 ± 0.01 g/cm^2^. Additionally, the thoracic spine BMD measured 0.83 ± 0.01 g/cm^2^. In comparison to individuals without MetS, patients with MetS exhibited significantly elevated levels of BMI, lumbar spine BMD, pelvic BMD, and thoracic spine BMD (all *p* < 0.05).

**Table 1:** Weighted baseline characteristics of participants categorized by the presence or absence of metabolic syndrome.

	Non-metabolic syndrome (n = 1099)	Metabolic syndrome (n = 825)	*P*-value
Age (year)	58.67 (0.32)	59.34 (0.36)	0.14
Body mass index (kg/m^2^)	26.96 (0.21)	32.14 (0.36)	** < 0.001**
Average calcium intake (mg)	940.02 (23.53)	887.92 (33.87)	0.26
Total cholesterol (mg/dl)	205.37 (1.58)	201.01 (1.85)	0.06
Low density lipoprotein (mg/dl)	123.25 (1.37)	117.85 (1.92)	**0.02**
LS-BMD (g/cm^2^)	1.00 (0.01)	1.04 (0.01)	** < 0.001**
Pelvic-BMD (g/cm^2^)	1.20 (0.01)	1.26 (0.01)	** < 0.001**
TS-BMD (g/cm^2^)	0.83 (0.01)	0.88 (0.01)	** < 0.001**
Sex (%)			0.46
Male	52.00	54.19	
Female	48.00	45.81	
Race (%)			0.14
Mexican American	4.38	5.98	
Non-Hispanic White	74.10	76.10	
Non-Hispanic Black	10.79	9.15	
Other Race	10.73	8.77	
Education Level (%)			**0.02**
Less than 9th grade	4.79	7.42	
9-11th grade	9.01	10.66	
High school graduate	23.81	28.75	
Some college or AA degree	29.06	29.52	
College graduate or above	33.33	23.65	
Marital status (%)			0.12
Married	64.08	64.08	
Widowed	6.80	8.00	
Divorced	15.42	14.30	
Separated	2.87	1.48	
Never married	5.97	8.88	
Living with partner	4.85	3.26	
Smoking status (%)			0.05
Never	46.79	43.08	
Former	31.34	38.35	
Now	21.87	18.57	
PIR (%)			**0.01**
Low-income	9.92	10.22	
Middle-income	43.79	51.89	
High-income	46.29	37.89	
Alcohol (%)			**0.003**
Former	17.27	23.61	
Heavy	13.96	16.16	
Mild	45.32	35.38	
Moderate	14.00	13.94	
Never	9.45	10.91	
Hypertension (%)			** < 0.001**
No	58.76	23.88	
Yes	41.24	76.12	
Diabetes (%)			** < 0.001**
No	92.39	63.13	
Yes	7.61	36.87	
Stroke (%)			**0.01**
No	96.65	93.63	
Yes	3.35	6.36	

All values are presented as proportion (%), or mean(standard error).

PIR, Ratio of family income to poverty; LS, lumbar spine; TS, ThoracicSpine; BMD, bone mineral density.

Significant values are in bold.

### Characteristics of participants stratified by sex

Table 2 presents the baseline characteristics of sex among study participants. The male group consisted of 1029 individuals with an average age of 58.37 ± 0.23 years and a calcium intake of 1021.06 ± 26.85 mg. The lumbar spine BMD was 1.05 ± 0.01 g/cm^2^, the pelvic BMD was 1.28 ± 0.01 g/cm^2^, and the thoracic spine BMD was 0.90 ± 0.01 g/cm^2^. The remaining 895 participants were female, with an average age of 59.60 ± 0.36 years. The calcium intake was 802.98 ± 19.30 mg. The lumbar spine BMD measurement was 0.98 ± 0.01 g/cm^2^, and the pelvic BMD measurement was 1.17 ± 0.01 g/cm^2^. Furthermore, the thoracic spine BMD measurement was 0.80 ± 0.01 g/cm^2^. Compared to females, males showed significantly higher levels of calcium intake, lumbar spine BMD, pelvic BMD, and thoracic spine BMD (all *p* < 0.05).

**Table 2:** Weighted baseline characteristics of participants categorized by sex.

	Male (n = 1029)	Female (n = 895)	*P*-value
Age (year)	58.37 (0.23)	59.60 (0.36)	** < 0.001**
Body mass index (kg/m^2^)	28.72 (0.23)	29.56 (0.27)	**0.01**
Average calcium intake (mg)	1021.06 (26.85)	802.98 (19.30)	** < 0.001**
Total cholesterol (mg/dl)	195.02 (1.55)	213.16 (1.83)	** < 0.001**
Low density lipoprotein (mg/dl)	117.02 (1.37)	125.50 (1.63)	**0.02**
LS-BMD (g/cm^2^)	1.05 (0.01)	0.98 (0.01)	** < 0.001**
Pelvic-BMD (g/cm^2^)	1.28 (0.01)	1.17 (0.01)	** < 0.001**
TS-BMD (g/cm^2^)	0.90 (0.01)	0.80 (0.01)	** < 0.001**
Sex (%)			0.46
Male	52.00	54.19	
Female	48.00	45.81	
Race (%)			0.48
Mexican American	5.31	4.75	
Non-Hispanic White	75.11	74.73	
Non-Hispanic Black	9.31	11.01	
Other Race	10.28	9.51	
Education Level (%)			**0.01**
Less than 9th grade	6.27	5.45	
9-11th grade	9.12	10.34	
High school graduate	23.98	27.96	
Some college or AA degree	28.18	30.46	
College graduate or above	32.45	25.79	
Marital status (%)			** < 0.001**
Married	68.98	58.58	
Widowed	2.92	12.22	
Divorced	12.86	17.31	
Separated	2.31	2.28	
Never married	8.16	6.08	
Living with partner	4.78	3.53	
Smoking status (%)			** < 0.001**
Never	37.43	54.04	
Former	39.02	28.89	
Now	23.55	17.07	
PIR (%)			0.07
Low-income	10.02	10.08	
Middle-income	44.25	50.41	
High-income	45.73	39.51	
Alcohol (%)			** < 0.001**
Former	19.77	20.05	
Heavy	18.76	10.51	
Mild	46.40	35.34	
Moderate	10.19	18.22	
Never	4.88	15.88	
Hypertension (%)			0.67
No	43.59	45.01	
Yes	56.41	54.99	
Diabetes (%)			0.06
No	78.31	82.38	
Yes	21.69	17.62	
Stroke (%)			**0.01**
No	96.06	94.65	
Yes	3.94	5.35	
Mets (%)			0.46
No	57.43	59.57	
Yes	42.57	40.43	

Significant values are in bold.

### The association between MetS and BMD

Tables 3 and 4 display the results of linear regression analyses that explored the association between MetS and BMD separately for postmenopausal females and males over 50 years. After fully adjusting for covariates, Table 3 indicates a positive correlation between MetS and pelvic BMD (β = 0.03, 95% CI 0.003–0.06) as well as thoracic spine BMD (β = 0.03, 95% CI 0.01–0.06). However, this correlation was not statistically significant for lumbar spine BMD (β = 0.020, 95% CI − 0.01–0.05). In Table 4, Model 2, after adjusting for age and ethnicity, a positive association between MetS and BMD at the pelvis (β: 0.046 [95% CI 0.02, 0.07]), thoracic spine (β: 0.047 [95% CI 0.02, 0.07]), and lumbar spine (β: 0.040 [95% CI 0.02, 0.06]) was observed. However, this relationship was not statistically significant in the fully adjusted model.

**Table 3:** Weighted multivariate linear regression models of metabolic syndrome with bone mineral density in men.

Bone mineral density	*β* (95% CI), *P*-value		
	Model 1^b^	Model 2^c^	Model 3^d^
LS-BMD (g/cm^2^)	0.035 (0.01, 0.07) **0.020**	0.040 (0.01, 0.07) **0.006**	0.005 (− 0.03, 0.04) 0.721
TS-BMD (g/cm^2^)	0.054 (0.03, 0.08) < **0.001**	0.060 (0.04, 0.08) < **0.001**	0.008 (− 0.01, 0.03) 0.484
Pelvic-BMD (g/cm^2^)	0.061 (0.03, 0.09) **0.002**	0.066 (0.04, 0.09) < **0.001**	0.020 (− 0.01, 0.05) 0.193

Model 1^b^: no covariates were adjusted; Model 2^c^: adjusted for age and race; Model 3^d^: adjusted for age, race, average calcium intake, education level, ratio of family income to poverty, marital status, body mass index, alcohol intake, smoking status, diabetes, hypertension, stroke, total cholesterol, and low density lipoprotein.

LS, lumbar spine; TS, thoracicSpine; BMD, bone mineral density; 95% CI,95% confidence interval.

Significant values are in bold.

**Table 4:** Weighted multivariate linear regression models of metabolic syndrome with bone mineral density in women.

Bone mineral density	*β* (95% CI), *P*-value		
	Model 1^b^	Model 2^c^	Model 3^d^
LS-BMD (g/cm^2^)	0.036 (0.01, 0.06) **0.006**	0.040 (0.02, 0.06) **0.002**	0.020 (− 0.01, 0.05) 0.238
TS-BMD (g/cm^2^)	0.046 (0.02, 0.07) < **0.001**	0.047 (0.02, 0.07) < **0.001**	0.030 (0.01, 0.06) **0.015**
Pelvic-BMD (g/cm^2^)	0.040 (0.02, 0.07) **0.002**	0.046 (0.02, 0.07) < **0.001**	0.030 (0.003, 0.06) **0.03**

Model 1^b^: no covariates were adjusted; Model 2^c^: adjusted for age and race; Model 3^d^: adjusted for age, race, average calcium intake, education level, ratio of family income to poverty, marital status, body mass index, alcohol intake, smoking status, diabetes, hypertension, stroke, total cholesterol, and low density lipoprotein.

LS, lumbar spine; TS, thoracicSpine; BMD, bone mineral density; 95% CI, 95% confidence interval.

Significant values are in bold.

### Subgroup analysis

To assess the robustness of our findings, subgroup analyses, and interaction tests were conducted, separating the data by gender to explore the potential impact of population stratification on the observed association between MetS and BMD (as shown in Supplementary Material 2–4). In male participants, analysis showed a consistent positive correlation between MetS and BMD across all age groups, with significant associations observed in both those under 65 and those 65 and older. A notable relationship was seen in individuals with a BMI under 30 kg/m^2^, but this association weakened and became non-significant for those with a BMI of 30 kg/m^2^ or higher. Smoking status also influenced the relationship, with current smokers demonstrating a stronger positive association than non-smokers, where the correlation was not significant. Moreover, a significant positive link between MetS and BMD was found regardless of hypertension status, with a notably stronger association in individuals with diabetes. For female participants, the analysis indicated a more pronounced association between MetS and BMD in the younger subgroup (< 65 years) compared to the older group (≥ 65 years). Similar to men, women with a BMI less than 30 kg/m^2^ showed a significant association, with a trend toward significance observed in those with a BMI of 30 kg/m^2^ or higher. Smoking status among females was consistently associated with BMD across all categories. The presence of hypertension did not impact the positive correlation between MetS and BMD, which remained significant across all hypertension statuses. Females with diabetes also displayed a strong positive association. Furthermore, interaction tests revealed that factors such as age, BMI, hypertension, stroke, and smoking status did not significantly affect the association (*p* for interaction > 0.05). However, a significant interaction was noted in the stratification by diabetes in the analyses of thoracic and lumbar spine BMD (*p* for interaction < 0.05).

## Discussion

Previous research has explored the relationship between MetS and BMD. For example, Kim et al., using data from the Korean National Health and Nutrition Examination Survey, assessed BMD associations across various demographic groups, including men of different ages and women who are pre- and postmenopausal^7^. However, based on Korean public databases, it may not be directly applicable to the U.S. population. A notable limitation is the use of a 45-year age threshold for male participants, thereby excluding those aged 50 and above. Furthermore, Kinjo et al.'s research, which utilized the NHANES III dataset from the U.S., is outdated and lacks focus on postmenopausal women and older men^32^. Additionally, while studies have investigated the correlation between MetS and BMD in adolescents using NHANES data, research on the elderly—a demographic particularly susceptible to BMD variations—remains sparse^43^. Our study seeks to fill this research gap by analyzing data from six NHANES cycles using weighted linear regression, underscoring the public health significance of focusing on this specific demographic. This cross-sectional study included 1029 participants and aimed to investigate the relationship between MetS and BMD, with a special emphasis on postmenopausal women and men over the age of 50. In postmenopausal women, MetS was found to significantly affect BMD elevation. In men, a significant impact of MetS on BMD was observed only after adjustments for age and ethnicity, indicating a relatively lower sensitivity to BMD changes compared to women. Subgroup analyses largely revealed a positive association between MetS and BMD, with diabetes identified as a key factor potentially affecting this relationship. Moreover, interaction tests showed that variables such as age, BMI, hypertension, stroke, and smoking status did not significantly alter this association.

This study focuses on two specific populations: males aged 50 and above and postmenopausal females. This focus arises due to the critical public health concern that bone density testing in these groups becomes with advancing age. In the United States, approximately one-third of postmenopausal women and one-fifth of males over 50 face an increased risk of fractures due to osteoporosis^44^. As age progresses, natural aging of the bones leads to decreased BMD, particularly in postmenopausal women, where a significant drop in estrogen levels further reduces BMD, significantly elevating fracture risk^45^. Previous investigations have explored the relationship between MetS and BMD within these unique populations. One study has identified gender differences in the risk of fractures associated with MetS^46^, noting an overall increase in BMD in the thoracic and lumbar spine and pelvis among MetS patients, more so in postmenopausal women^47^. Research in non-diabetic adults in the United States demonstrated a positive correlation between the Metabolic Syndrome Insulin Resistance (METS-IR) score and BMD levels, revealing that an increase in METS-IR by one unit significantly enhances total femoral and spinal BMD^48^. This underscores the importance of considering metabolic factors in BMD assessment, especially in populations at risk of MetS, including males over 50 and postmenopausal females. While some studies did not specifically focus on these distinct groups, they have corroborated the positive correlation between MetS and BMD. Research indicates that abdominal obesity may increase mechanical stress on bones, leading the body to augment BMD by increasing bone accumulation, particularly in the context of abdominal obesity^49^. Additionally, the study by Jiang et al. provided substantial evidence of a consistent relationship between low levels of HDL-C and an increase in BMD in the context of MetS while also indicating that elevated triglyceride levels positively correlate with BMD^50^. Reports have suggested that individuals with insulin resistance and elevated insulin levels due to MetS might experience an increase in BMD^51^. Animal experiments further corroborated these findings, demonstrating that insulin could enhance BMD by facilitating osteocalcin signaling, aligning with our study's conclusions^52^.

The exact mechanism linking MetS and BMD is yet to be fully understood. It is hypothesized that MetS might lead to changes in biochemical profiles, possibly by affecting hormone regulation and the function of adipokine^53^. Particularly in menopausal women, MetS has been noted to slow down the reduction of estrogen levels in the body^54^. A wealth of research supports the crucial role of estrogen in human bone metabolism, illustrating its influence on osteoclast activity and thereby inducing alterations in BMD through multiple mechanisms^55^. Initially, estrogen deficiency has been identified as a factor that increases the permeability of the intestinal epithelium, thereby facilitating the entry of intestinal pathogens and initiating an immune response^56^. This immune reaction leads to heightened bone resorption by osteoclasts and a subsequent decrease in BMD^57,58^. Moreover, the lack of estrogen impedes the production of IL-1 and Tumor Necrosis Factor (TNF)^59^. Notably, IL-6 and TNF-alpha, stimulated by IL-1 and TNF, respectively, incite an inflammatory response that encourages osteoclast formation and their bone-resorbing activities^60^. This cascade results in osteolysis, bone loss, and an elevated calcium concentration in the bloodstream, contributing to a decline in BMD^61^. Another study has shown that MetS can enhance BMD by regulating adipokine secretion^62^. MetS activates the Wnt signaling pathway, renowned for its osteogenic capabilities^63^, and affects BMD by altering gene expression related to adipokine secretion in adipocytes^64^. Leptin, a peptide hormone from adipocytes, plays a pivotal role in enhancing osteoblast activity and suppressing osteoclast formation through the RANKL/OPG pathway, thus obstructing osteoclastogenesis^49^. Leptin levels are positively associated with BMD, especially in menopausal women^65^. Additionally, vaspin, a novel adipokine from visceral fat, has been shown to foster bone formation by protecting osteoblasts and preventing bone erosion by inhibiting osteoclasts^66^. On the contrary, Omentin-1, another adipokine from visceral fat, may exhibit anti-inflammatory qualities and counteract the pro-osteoclastogenic effects triggered by macrophage activation^67^. It's important to note that MetS patients show biochemical changes that might influence BMD modulation. In particular, alterations in HDL-C concentrations and blood glucose levels have been recorded^68^. MetS often correlates with decreased HDL-C levels, and research has consistently shown a negative relationship between HDL-C levels and BMD^69^. This link is likely due to the pro-inflammatory response induced by high HDL-C levels, which in turn boosts osteoclast activity, ultimately reducing BMD. Additionally, hyperglycemia, a known risk factor associated with MetS, may affect bone metabolism^70^. People with MetS frequently have high insulin levels^71^, and studies suggest that insulin signaling in osteoblasts reduces osteoprotegerin expression, a suppressor of osteoclast formation^72^. This action promotes bone resorption, leading to an increase in BMD^73–75^.

Our subgroup analysis further reveals that the positive correlation between MetS and BMD may be more pronounced in the diabetic population. The impact of diabetes on BMD cannot be overlooked, yet the precise mechanisms behind it have not been fully elucidated to date. Current research indicates that the maintenance of bone health heavily relies on normal glucose metabolism^76^, with the differentiation and function of osteoblasts largely dependent on glucose supply^77^. On this basis, the elevated blood glucose levels in diabetic patients theoretically provide more glucose for the activity of osteoblasts, which may promote an increase in bone density. Moreover, the application of anti-diabetic medications also has a direct or indirect impact on BMD^78^. For instance, metformin, a widely used anti-diabetic drug, may promote an increase in BMD by regulating the expression of key osteoblast markers—core binding factor A1 and LDL receptor-related protein 5^79^. Further studies have pointed out that being overweight and hyperinsulinemia, as two hallmark features of type 2 diabetes, are closely related to a positive correlation with BMD^80^. From a physiological perspective, insulin can exert an anabolic metabolic effect on bones by interacting with the IGF-1 receptor on the surface of osteoblasts^81^. Moreover, the IGF-1 signaling pathway is crucial for the healthy growth of bones^82^, further substantiating the viewpoint that hyperinsulinemia positively correlates with BMD. Another noteworthy finding is that in male diabetic patients, the concentration of leptin in plasma is higher than in the healthy control group^83^. Leptin has been proven in vitro to promote an increase in BMD by stimulating the proliferation and differentiation of osteoblasts^84^. These research outcomes not only enrich our understanding of the relationship between diabetes and BMD but also provide important scientific bases for future therapeutic strategies aimed at enhancing BMD.

Our study boasts several notable strengths. Firstly, it utilizes data from the respected NHANES, which has been weighted to accurately reflect the correlation between MetS and BMD among postmenopausal women and men over 50 across the United States. Additionally, we have addressed confounding variables, selecting these based on insights from prior research, which bolsters the credibility of our results. Crucially, this research holds significant public health importance by pinpointing populations at increased risk of bone density changes, laying the groundwork for targeted public health interventions. In an aging society, comprehending how MetS influences BMD, especially in high-risk groups, is essential for the prevention of associated conditions, including osteoporosis.

However, it is imperative to acknowledge specific limitations inherent in our study. Firstly, the utilization of cross-sectional data from the NHANES restricts our capacity to establish a definitive causal relationship between MetS and BMD. What’s more, despite our meticulous endeavors to control for potential confounding variables, it is crucial to recognize that the impact of unmeasured or residual confounders cannot be completely eradicated.

## Conclusions

This study has elucidated the complex relationship between MetS and BMD in a gender-specific context among individuals over the age of 50. Our findings reveal that in postmenopausal women, a significant positive correlation exists between MetS and BMD at the pelvis and thoracic spine after adjusting for all covariates, a correlation not observed for the lumbar spine BMD. In contrast, for males, initial analyses suggested positive correlations between MetS and BMD at the lumbar spine, thoracic spine, and pelvis in models adjusting for age and ethnicity. However, these correlations were not sustained upon full adjustment for all covariates. The gender-specific impact of MetS on BMD underscores the need for gender-informed clinical approaches and a reevaluation of guidelines for osteoporosis and MetS management. Future research should focus on understanding the biological and lifestyle factors driving gender differences in the MetS–BMD relationship, aiming to develop targeted interventions.

### Supplementary Information

Supplementary Information.

## Data Availability

The survey data are publicly available on the Internet for data users and researchers throughout the world (www.cdc.gov/nchs/nhanes/).
